# Energetically optimal running requires torques about the centre of mass

**DOI:** 10.1098/rsif.2012.0145

**Published:** 2012-04-04

**Authors:** James R. Usherwood, Tatjana Y. Hubel

**Affiliations:** Structure and Motion Laboratory, The Royal Veterinary College, University of London, North Mymms, Hatfield, Herts AL9 7TA, UK

**Keywords:** run, hop, spring-mass, point mass, ground reaction force

## Abstract

Bipedal animals experience ground reaction forces (GRFs) that pass close to the centre of mass (CoM) throughout stance, first decelerating the body, then re-accelerating it during the second half of stance. This results in fluctuations in kinetic energy, requiring mechanical work from the muscles. However, here we show analytically that, in extreme cases (with a very large body pitch moment of inertia), continuous alignment of the GRF through the CoM requires greater mechanical work than a maintained vertical force; we show numerically that GRFs passing between CoM and vertical throughout stance are energetically favourable under realistic conditions; and demonstrate that the magnitude, if not the precise form, of actual CoM-torque profiles in running is broadly consistent with simple mechanical work minimization for humans with appropriate pitch moment of inertia. While the potential energetic savings of CoM-torque support strategies are small (a few per cent) over the range of human running, their importance increases dramatically at high speeds and stance angles. Fast, compliant runners or hoppers would benefit considerably from GRFs more vertical than the zero-CoM-torque strategy, especially with bodies of high pitch moment of inertia—suggesting a novel advantage to kangaroos of their peculiar long-head/long-tail structure.

## 1. Introduction

Legs of running or hopping animals not only support body weight, but also act to slow and accelerate the body horizontally over the course of every step. The resulting fluctuations in kinetic energy—which must impose some degree of energetic cost at the level of the muscle owing to the absence of perfectly elastic springs in biology—are perhaps perplexing. Why ‘should’ such wasteful fluctuations in kinetic energy be suffered, if the purpose of steady, level gaits is economic locomotion? Alexander [1] approached this issue analytically, considering the work requirements of functional knee and hip for two extreme cases: forces maintained through the centre of mass (CoM) and purely vertical forces. The combined positive work requirements of the two joints are smaller in the first case—and an elegant account is made on energetic grounds for the empirical observation that GRFs in running and hopping animals pass approximately through the CoM.

Alexander's analysis assumes that the work requirements of the functional ‘hip’ and ‘knee’ can be treated separately and simply added. However, this need not be the case: simple mechanisms, such as two-joint muscles, allow energy ‘lost’ at one joint to be apparently ‘gained’ at another, without net mechanical work [2], though the actual efficacy of such mechanisms is uncertain [3–5].

We first demonstrate analytically that if the opposite assumptions are adopted concerning between-joint energy transfer, the opposite prediction is made. If *only* the net work performed by the entire leg ‘costs’, stances with purely vertical forces are more economical than those with GRFs maintained through the CoM.

We then consider the effects of realistic vertical forces and pitch moments of inertia *I*_pitch_, incorporating the effects of pitching motions of the body, and explore the energetic consequences of a range of GRF alignments. Predicted CoM-torque profiles are compared with observed, and further implications of body form (*I*_pitch_) and gait (speed *V* and duty factor, the proportion of stride spent in stance, *β*) are discussed.

## 2. Maths and methods

### 2.1. Analytical demonstration that through centre of mass forces are not always energetically optimal

**Figure 1.**
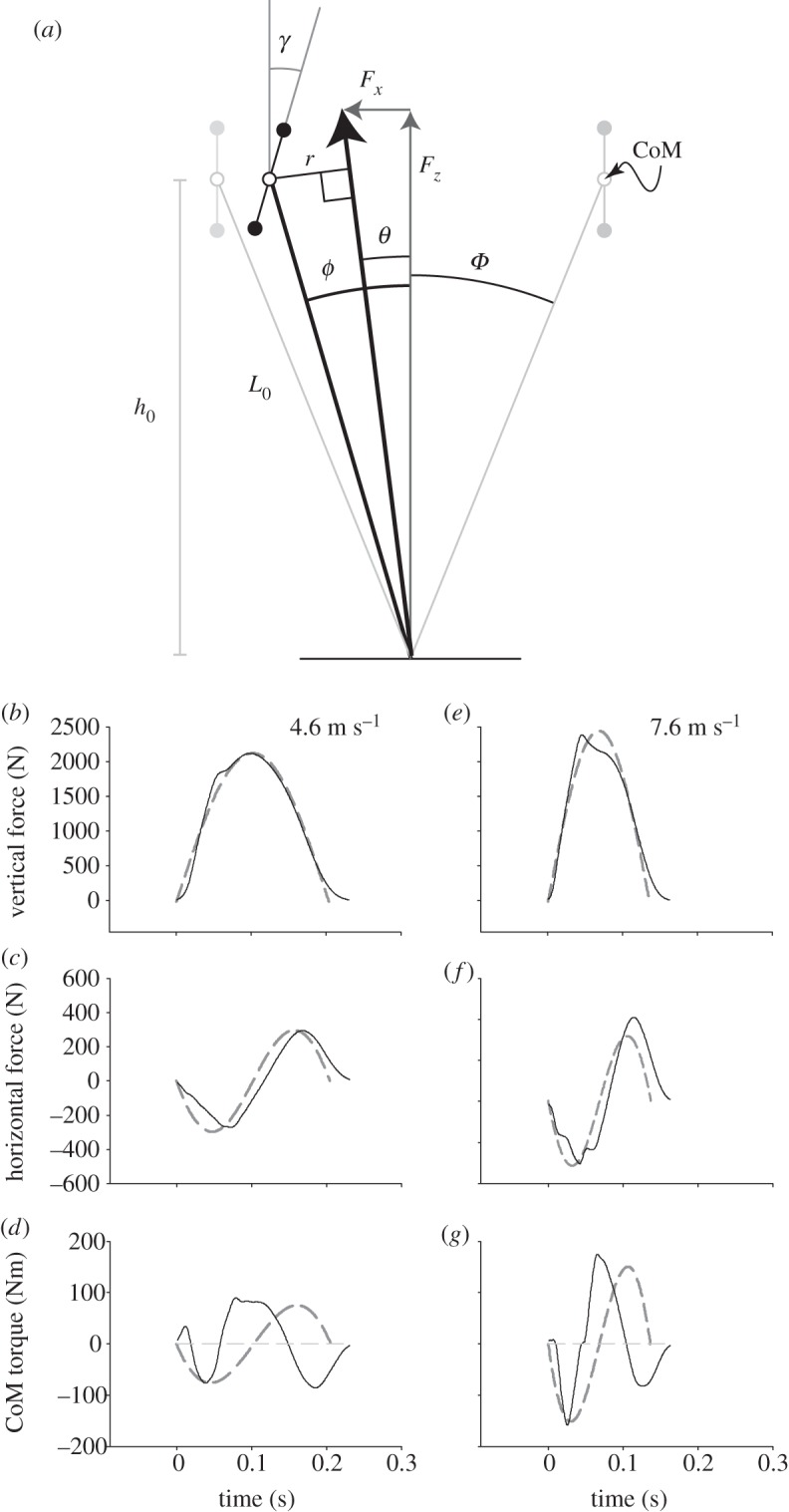
(*a*) The geometry and (*b–g*) results of a model (dashed grey curves) using half-sine approximations of measured vertical GRFs (black lines) for running humans based on mechanical work minimization. While the optimal GRFs (bold black arrow, *a*) pass close to the CoM, they actually pass closer to vertical, thereby reducing fluctuations in horizontal kinetic energy, albeit at a cost in terms of rotational kinetic energy. Thus, unlike point-mass models or zero-torque assumptions (pale grey horizontal dashed line, *d,g*) torques about the CoM are predicted. These are observed (*d*,*g*) with appropriate magnitude, but with no account for the consistently observed second inflection (black lines, *d*,*g*).

Consider two extreme strategies for providing weight support over a symmetrical stance passing from −*Φ* to +*Φ* (figure 1*a*). The first is totally ‘telescoping’, with forces always in line with the CoM and no CoM-torques. The second has purely vertical forces; there are CoM-torques but never horizontal forces. In the two cases, the vertical force profile—and hence related vertical work requirements—are assumed to be equivalent.

#### 2.1.1. The horizontal work of supporting a body without torques about the centre of mass

The horizontal force *F_x_* required to keep the GRF orientated through the CoM is2.1
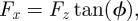
where *ϕ* is the angle of the GRF from vertical. The horizontal deflection d*x* with a motion over a small angle d*ϕ* is2.2
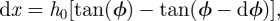
where the initial height *h*_0_ depends on initial leg length *L*_0_2.3
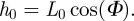
Integrating the product of force and deflection over the stance gives the horizontal work input required for this ‘zero-torque’ strategy 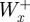 over a step2.4
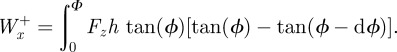


Note that the positive part of this work occurs after midstance (*ϕ* > 0) which is why the integration begins with a vertical leg (*ϕ* = 0).

#### 2.1.2. The torque-work of supporting a body with no horizontal forces and very large pitch moment of inertia

In this case, the GRF is always orientated vertically, resulting in no horizontal energy fluctuation, but generating torques about the CoM. The moment arm *r* acting about the CoM (figure 1*a*) due to the vertical force is2.5
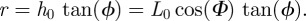
The resulting torque *Q* is given by2.6
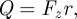
and the torque-work d*W*_torque_ performed for a small change in angle d*ϕ* is2.7
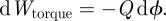


For this limiting analytical example, we consider the case where *I*_pitch_ is sufficiently large to prevent any pitching of the body. The total positive torque-work required for supporting weight by this purely vertical force strategy 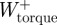 is given by2.8
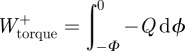


and2.9
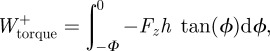
noting that, in this case, the positive works occur in the first half of stance.

#### 2.1.3. Telescope or torque-power?

Which strategy for supporting body weight is energetically favourable? The similarity of expressions for work requirements (in addition to vertical, equations (2.4) and (2.9)) makes the comparison simple: the through-the-CoM, zero CoM-torque strategy is energetically superior if2.10
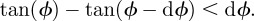
However, this is never the case (they converge as |*ϕ*| → 0). This analysis therefore demonstrates that, with a distributed mass in the sagittal plane *and perfect energy interchange between leg joints*, non-zero CoM-torques offer an energetic advantage for supporting body weight in running or hopping.

### 2.2. Numerical modelling of weight support strategies

In order to model the energetic consequences of a range of weight support strategies during running, we (i) assume vertical ground reaction forces (GRFs) to be half-sinusoidal (we use fits to observed GRFs over a range of speeds, figure 1*b,e*), (ii) use measured height and body mass, and isometrically scaled *I*_pitch_ (for the ‘head, arms and trunk’ (HAT) for an 80 kg male [5] about the HAT CoM), and (iii) calculate the energetic consequences of a range of weight support strategies, from purely through the CoM to purely vertical. The range of support strategies used here is not a thorough search of the parameter space. We merely keep the ratio of GRF angle (*θ*, figure 1*a*) to foot-CoM angle (*ϕ*) constant throughout stance: when the ratio is 1, the force goes through the CoM, and there are no CoM-torques; when zero, the force is vertical throughout stance.

Three forms of mechanical power are calculated separately: vertical (which is consistent for a given vertical force profile), horizontal (highest if the GRF passes through the CoM, lowest if it is held vertical) and pitch. The pitch power *P_Q_* depends on: the torque about the CoM *Q* (treated here as effectively the ‘hips’, but *Q* will not be exactly the same as hip torques derived from inverse dynamics); the angular velocity of stance leg retraction d*ϕ*/d*t*; and the angular velocity of body pitching (d*γ*/d*t* (figure 1*a*), which must be zero for infinite pitch moment of inertia):2.11
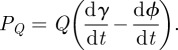
We search for the *θ*/*ϕ* ratio that results in the lowest positive mechanical work.

It should be noted that we do not include any ‘energy transfer’ between the rotational kinetic energy of pitching and the translational kinetic energies of the CoM. While such transfer is observed in brachiating animals [6] where the limb is rotating in the same sense as the body (and a simple ‘braking’ mechanisms can be imagined), leg and body rotation senses in running are, at least according to the model, opposite at midstance. We therefore assume that there is no mechanism for returning ‘rotational’ kinetic energy to ‘translational’ for runners.

### 2.3. Empirical measurements of centre of mass torques in running humans

Four fit, adult men ran at speeds ranging from slow to near-maximal over a series of eight Kistler 9287B forceplates recording GRFs at 500 Hz. Speeds were determined from motion capture (eight Qualysis Oqus cameras). Only steady trials are included in analysis (average accelerations under 0.2 ms^−2^). CoM motions and subsequent calculation of moments about the CoM during each stance were derived from forceplate measurements [7] with the following assumptions for each complete step: no change in vertical or horizontal speed; no change in height; zero net torque. This last assumption is not widely used but, with the anatomical observation that the CoM lies at 58.4 per cent of body height [8], allows the absolute position of the CoM in the sagittal plane to be calculated—and allows calculation of CoM-torques in pitch throughout the stance.

## 3. Results and discussion

**Figure 2.**
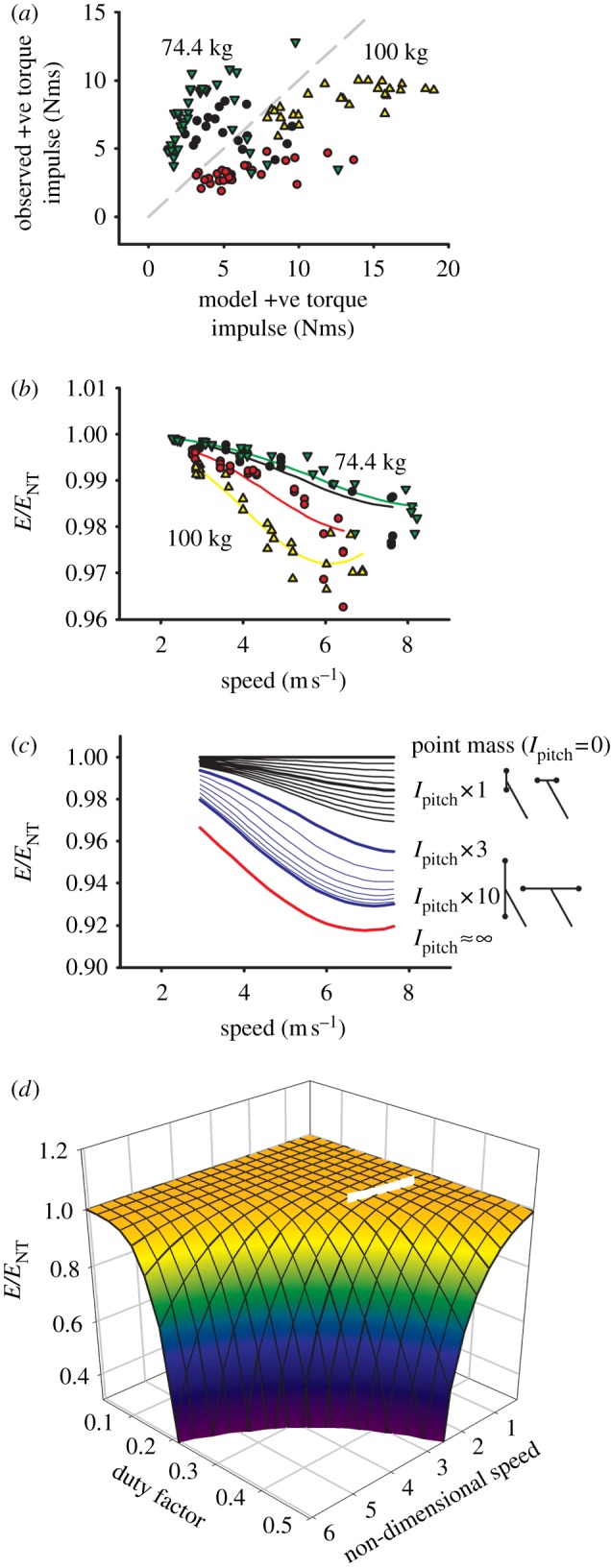
The model provides an encouraging, though far from precise, prediction (dashed line) of the torque impulses about the CoM observed in four humans (denoted by symbol, *a,b*) running at a range of speeds (*a*). (*b*) The modelled energetic cost of torqueing weight support as a proportion of the zero-CoM-torque strategy (*E*_NT_). These could be further reduced with increased pitch moment of inertia (*c*), based on the values of a subject of intermediate mass (black line in (*b*)). At higher speeds and duty factors, the potential savings (using human *I*_pitch_ properties) become considerable (*d*, with the human trajectory shown as a white line on the surface).

Measured vertical GRFs in running humans are close to half-sinusoidal, with maxima increasing with reduced duty factor (proportion of stride in stance for a given leg) associated with increasing speed (e.g. grey lines, figure 1*b*,*e*). Measured horizontal GRFs approximate full (negative) sine-waves, though less closely [9]. Horizontal forces predicted from numerical energy minimization (figure 1*c*,*f*) are constrained to being close to negative sinusoidal because of the limited parameter space searched (*θ*/*ϕ* ratios constant throughout stance). Measured torques about the CoM (black lines, figure 1*d*,*g*) were of a consistent general shape, increasing in amplitude with speed. While some aspects of the observed torque waveform—the impulse (figure 2*a*), the pitch-backward tendency in early stance followed by a pitch-forward torque—match the predictions of energy minimization, the second inflection of the profile towards the end of stance is not. As yet, we have no account for this observation: it may be due to the limited parameter space searched; or limitations of the mechanical model—a prime candidate being the inertia of the swing legs; or, of course, the premise of work minimization itself may be incorrect. However, our exceedingly simple reduction of a runner (a telescoping/torqueing massless leg connected to a body of appropriate pitch inertia) and energy minimization predicts CoM-torques (i.e. GRFs *not* aligned through the CoM), perhaps surprisingly, at close to observed magnitudes (figure 2*a*).

But how relevant is the energy saving produced by CoM-torques? Comparing the optimized model work (*E*, based on the half-sine vertical GRF, using the best *θ*/*ϕ*) with the ‘no-torque’ work required *E*_NT_ *if* the force went directly through the CoM throughout stance (*θ*/*ϕ* = 1), suggests that the savings can approach 4 per cent (figure 2*b*).

The model allows some consideration of the implications of body form. For a given range of vertical force profiles, what energy might be saved with a range of pitch moments of inertia? A point-mass body cannot impose or apply torques; *E/E*_NT_ must equal 1. With increasing pitch moments of inertia, optimal GRFs become more vertical, but the potential savings (even with infinite moment of inertia and constantly vertical GRFs) are limited—approximately 8 per cent for a human (figure 2*c*).

The implications of basic kinematic parameters can also be modelled. Assuming the half-sine relationship for vertical GRF holds over the full range of duty factors, the potential energetic savings can be presented conveniently using non-dimensional speed 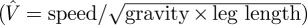 (figure 2*d*). The potential savings from non-zero-torque (more vertical) GRFs increase dramatically with speed and duty factor. This is because, at high speeds and stance angles, horizontal kinetic energy fluctuations become disproportionately high. Indeed, at the (albeit unrealistic) limiting case where the stance length equals twice the leg length (a biped does the ‘splits’ each step), locomotion can *only* be achieved with CoM-torques. This limiting condition occurs at3.1
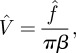
where 
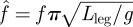
 is the step frequency normalized by natural frequency of a pendulum of leg length. Exceeding this speed/duty factor boundary is geometrically impossible—hence the excluded region in figure 2*d*.

Therefore, in cases where high speeds and high duty factors are observed, greater benefit from torque-strategy weight support would be predicted. And the energetic savings of such torqueing strategies would be further enhanced with an increased pitch moment of inertia. This suggests an intriguing, novel mechanical account for the long-tail/long-neck structure of kangaroos. While elements within the neck and the tail are undoubtedly somewhat elastic, and deflect considerably during hopping [10] (thus potentially obscuring any pitching motions—body pitch amplitudes in this study were less than 5°) the high pitch moment of inertia ‘T’-shaped structure of these fast, high stance-angle hoppers might best be understood as a means to increase the pitch moment of inertia in order to take better advantage of torqueing weight support strategies.

## 4. Conclusion

Torques about the CoM during stance can—with non-zero body pitch moments of inertia—reduce the mechanical work of supporting body weight by reducing horizontal forces, and thus fore–aft energy fluctuations. We therefore propose an energetic—rather than stability [11]—account for relatively vertical GRFs. The potential for energetic savings is increased with higher pitch moments of inertia, higher speeds and higher duty factors.
